# Consideration of Thermo-Vacuum Stability of a MEMS Gyroscope for Space Applications

**DOI:** 10.3390/s20247172

**Published:** 2020-12-15

**Authors:** Jili Liu, Mingrui Fu, Chao Meng, Jianpeng Li, Kai Li, Jun Hu, Xiaojuan Chen

**Affiliations:** Science and Technology on Space Intelligent Control Laboratory, Beijing Institute of Control Engineering, Beijing 100194, China; fumingrui@c502.cast.casc (M.F.); mengchao@c502.cast.casc (C.M.); lijianpeng2@c502.cast.casc (J.L.); likai@c502.cast.casc (K.L.); hujun@c502.cast.casc (J.H.); chenxiaojuan@c502.cast.casc (X.C.)

**Keywords:** MEMS gyroscopes, thermo-vacuum stability, bias drifts, space applications, sensor, self-compensation

## Abstract

Thermo-vacuum stability of the aerospace gyroscopes is one of the crucial issues in the harsh and remote environment of space. This paper reports a bias drift compensation algorithm for the MEMS (microelectromechanical systems) gyroscope with atmosphere package. This approach takes advantage of linear frequency–temperature dependence and linear amplitude–pressure dependence for self-compensation of the gyroscope bias drifts in real-time. The dependences were analyzed and evaluated by subjecting the gyroscope to a thermo-vacuum condition. The real-time self-compensation yielded a total bias error of 0.01°/s over a temperature range of 7–45 °C. A MEMS rate sensor was flown in space and the on-orbit data also verify the effectiveness of the approach.

## 1. Introduction

Over the past two decades, MEMS gyroscopes are widely used in consumer electronics, automotive safety, robotics, and stabilization due to their small size and low power consumption [1,2]. With improved performance, they also have the great potential to enable more advanced applications, like space applications. For this reason, MEMS gyros are promising for failure detection in large satellites or for attitude propagation and rate determination in microsatellites, telecom satellites and planet rovers [3,4,5].

Nevertheless, in spite of their improved performance, the MEMS gyroscopes still require major technical adaptations to be suitable for the space applications, especially the high resistance characteristics with respect to the aerospace environment. A great number of research works have been devoted to the field of the MEMS reliability. Commonly, most of the space-specific reliability concerns are thermal cycling and thermal shocks, radiation, vibration and mechanical shock at launch and stage/heat shield separation [6,7,8,9].

The life of the microsatellite is mostly one year. On the one hand, the gyroscope is required to have the best cost, size, weight and power (CSWaP). On the other hand, the gyroscope should work stably during the service life of the satellite. The atmosphere-packaged MEMS gyroscope is one of the best candidates due to its cost advantage. However, the high vacuum of the space environment is a factor that cannot be ignored for the MEMS gyroscopes with atmosphere package. The air pressure in the gyroscope package will drop over a period of time in the very high vacuum condition. The bias drift of the MEMS gyroscope is related to the operating pressure [10]. Another bias drift source for MEMS gyroscopes is their inherent sensitivity to the temperature variation [11]. Consequently, engineers shall pay adequate attention to sensitivity of the gyroscope bias towards thermo-vacuum environment.

For the purpose of enhancing the reliability of the atmosphere-packaged MEMS gyroscope for the use in space, a MEMS gyroscope with two vibrating masses is used in this paper. To achieve this goal, the factors causing the gyroscope bias drift are analyzed, especially when the contribution of the temperature and the air pressure in the gyroscope package to the total bias drift is investigated. With the help of a vacuum chamber, the relationship between temperature and bias drift, as well as the relationship between the air pressure and bias drift is characterized. On this basis, a novel control system using the drive-mode parameters for the MEMS gyroscope bias compensation is proposed. In order to verify the effectiveness of the compensation algorithm, a MEMS rate sensor based on these atmosphere-packaged MEMS gyroscopes is developed and adopted for the on-orbit technology demonstration.

This paper is organized as follows. Section 2 presents an analysis of thermo-vacuum condition induced bias drift in the MEMS gyroscope, and then an approach for self-compensation of the gyroscope bias drifts is demonstrated. In Section 3, the effectiveness of the approach is validated using the experimental data and the on-orbit data respectively. We draw some conclusions in Section 4.

## 2. Bias Drift Analysis and Compensation

The bias of MEMS gyroscope (B) can be described as [10,12]:

(1) $$B=\frac{1}{\mathrm{nk}}\left(\frac{1}{\tau_{1}}-\frac{1}{\tau_{2}}\right)\sin\left(\theta_{\tau }\right)$$

where n is the vibratory mode number (which is usually equal to 2), k is the angular-gain factor (which depends on the geometry of the sensor and is very stable over temperature and lifetime of the device), $\frac{1}{\tau_{1}}$, $\frac{1}{\tau_{2}}$ are the principal damping values, $\theta_{\tau }$ is the principal axis (drive-mode) of damping. Equation (1) can be rewritten as:

(2) $$B=\frac{1}{2k}\Delta \left(\frac{1}{\tau }\right)\sin\left(\theta_{\tau }\right)$$

where $\Delta \left(\frac{1}{\tau }\right)$ is the damping mismatch between vibratory modes, $\sin\left(\theta_{\tau }\right)$ is a sine function with an absolute value less than 1, thus the upper bound of Equation (2) can be written as follows:

(3) $$B=\frac{1}{2k}\Delta \left(\frac{1}{\tau }\right)\sin\left(\theta_{\tau }\right)\leq \frac{1}{2k}\Delta \left(\frac{1}{\tau }\right)$$

We can see that the rate bias of the gyroscope is bounded by the difference in principal axis damping mechanisms. Air-damping, thermoelastic dissipation, and anchor and surface losses are the most well-known energy loss mechanisms in silicon-based MEMS gyroscopes [13]. Among these damping mechanisms, the air-damping is dominant, especially for the gyroscope with atmosphere package. Any variation of the air pressure in the gyroscope package will lead to a change of the air damping, and thus the drift of the gyroscope bias. Fabrication and packaging imperfections, as well as electronic component drifts, may also contribute to change in the angle $\theta_{\tau }$ of the principal axis of damping, and thus overall temperature sensitivity of the gyroscope bias.

**Figure 1:**
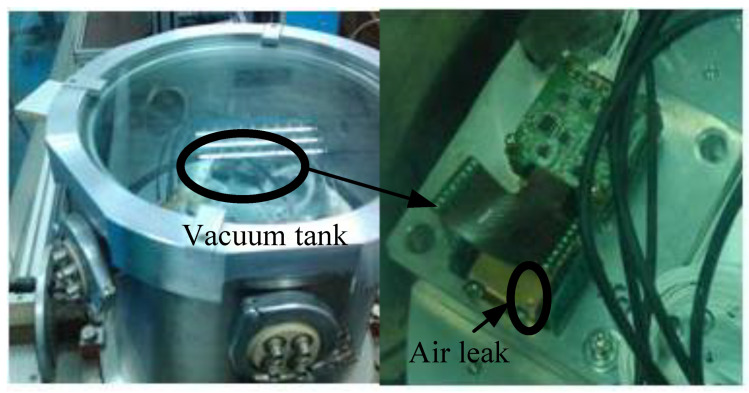
Photo of the experimental setup, showing the vacuum chamber and the MEMS gyroscope with an air leak.

In order to figure out how temperature and the air pressure affect the gyro’s bias, a vacuum chamber is used to create a thermo-vacuum environment, as shown in Figure 1. It is worth noting that a small hole was deliberately drilled in the MEMS gyroscope. The existence of the air leak makes it possible to change the air pressure in the gyroscope package with the assistance of the vacuum chamber. Thus, the effects of the temperature and the air pressure on the gyroscope bias can be revealed separately.

### 2.1. Temperature-Induced Bias Drift

**Figure 2:**
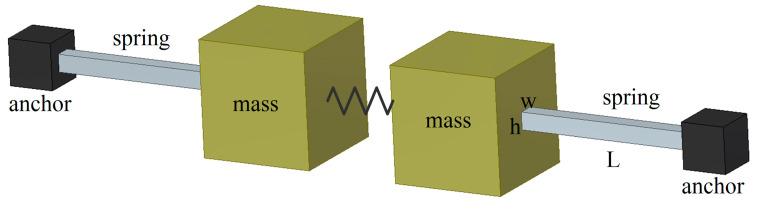
Simplified spring-mass structure.

The MEMS gyroscope is featured with the typical spring-mass structure [14], as shown in Figure 2.

The frequency is determined by the physical parameters of the spring, and the resonator frequency of the drive mode can be expressed as follows [15,16,17,18]:

(4) $$w_{d}=\frac{1}{2\pi }\sqrt{\frac{{4\mathrm{EhW}}^{3}+{\pi \mathrm{AL}}^{2}\sigma /4}{{\mathrm{mL}}^{3}}}$$

where E is the Young’s modulus, m is the effective mass of the gyroscope, h, W, L are the mechanical parameters of the gyroscope spring, A is the cross-section of the spring (A=h*W), σ is the residual stress. Equation (4) can also be simplified as follows:

(5) $$w_{d}=\lambda \sqrt{1+\eta E}$$

where λ and η are used and they are both constants:

(6) $$\{\begin{array}{c} \lambda =\frac{{\mathrm{AL}}^{2}\sigma }{8L\sqrt{\mathrm{mL}}}\\ \eta =\frac{{16\mathrm{hW}}^{3}}{{\pi \mathrm{AL}}^{2}\sigma } \end{array}$$

The variations of the mechanical parameters induced by the temperature change can be negligible compared to that of the Young’s modulus. Thus, Equation (5) can be rewritten as:

(7) $$w_{d}\approx \lambda \sqrt{1+\eta \left(E_{0}+\Delta E\right)}=\lambda \sqrt{1+{\eta E}_{0}}\sqrt{1+\frac{\eta \Delta E}{1+{\eta E}_{0}}}$$

where $E_{0}$ is the Young’s modulus at the room temperature, ΔE is the variation of the Young’s modulus due to a change in the temperature. For the silicon, the variation of the Young’s modulus changes linearly over a small temperature range [19]. The variation of the Young’s modulus is a very small value compared to $E_{0}$, so Equation (7) can be approximately described as:

(8) $$w_{d}\approx \lambda \sqrt{1+{\eta E}_{0}}\left(1+\frac{1}{2}\frac{\eta \Delta E}{1+{\eta E}_{0}}\right)$$

As can be seen from Equation (8), the silicon resonator frequency changes linearly with the variation of the Young’s modulus, or the temperature. So, the drive-mode frequency is usually used as a built-in thermometer. The temperature coefficient of the drive-mode frequency is well defined by elastic properties and its value is fixed for a given material (e.g., silicon).

The frequency–temperature dependence was experimentally measured over a temperature range of −20–50 °C. Table 1 lists the drive-mode frequencies at various temperatures. During the experiment, each temperature was kept for at least 2 h in order to cool, or heat the gyroscope through.

The temperature coefficient of the drive-mode frequency (TCF) was obtained by fitting the data in Table 1. The result revealed a negative correlation coefficient of −0.1 Hz/°C. 

The relationship between the temperature and the gyroscope bias was exposed by experiment. During the experiment, the air pressure in the vacuum chamber was pumped below 1 × 10^−4^ Pa. Thus, the bias drift due to the air pressure effect was largely eliminated. In the near vacuum, the gyroscope with an air leak suffered from a temperature change from −25 °C to 40 °C.

**Figure 3:**
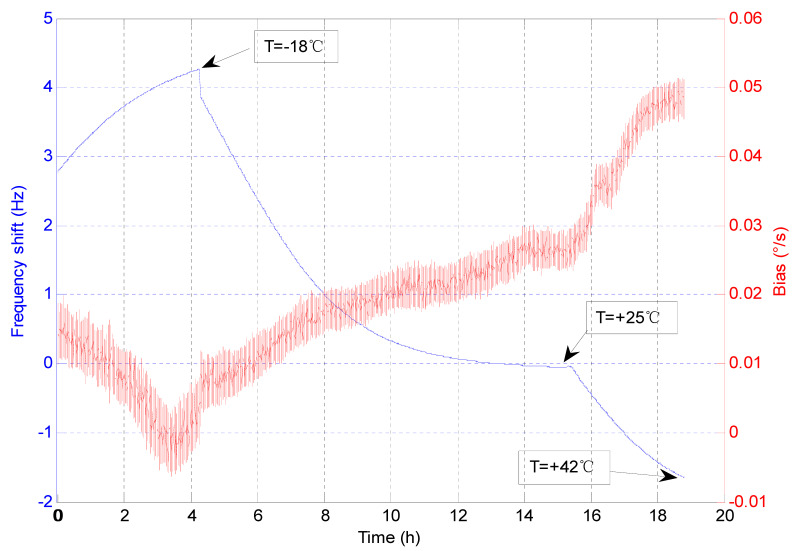
Measured bias drift vs. frequency shift.

During a 19-h run, the inverse relationship was observed for the gyroscope bias drift as a function of the drive-mode frequency shift. Here, the drive-mode frequency at the temperature of 25 °C was used as the reference. The actual temperature values marked in Figure 3 were obtained by monitoring the gyroscope drive-mode frequency and using the linear TCF value for the conversion. Due to the poor thermal conductivity in the vacuum, the actual temperatures of the MEMS gyroscope are usually higher than that of the vacuum environment.

According to the linear least squares fit to the experimental data, a bias-frequency dependence of −0.0097 (°/s)/Hz was obtained.

### 2.2. Air Pressure-Induced Bias Drift

**Figure 4:**
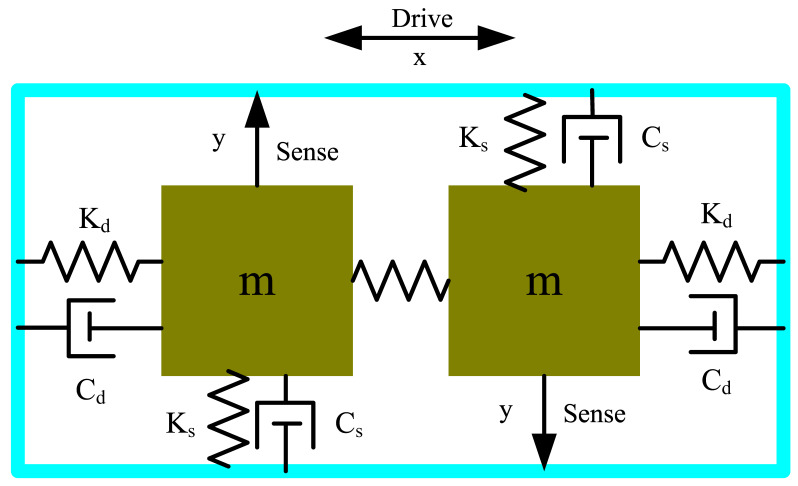
Dual mass gyroscope modelled as spring-mass-damper system.

The MEMS gyroscope used in this work can also be modelled as spring-mass-damper system with two vibrating masses. If an angular rate around the z-axis is applied to this type of sensor, the masses m will be deflected in the positive and negative y-direction, as is shown in Figure 4. To achieve a stable oscillation, the masses m are driven in a resonant anti-phase mode. A resonant oscillation in anti-phase is beneficial due to inherent common-mode rejection.

The kinetic equation of the MEMS gyroscope can be obtained if the drive signal is set to $F_{0}{\sin(w}_{f}t)$ [20]:

(9) $$\{\begin{array}{c} {mx}^{\prime \prime }+C_{d}x^{\prime }+K_{d}x=F_{0}{\sin(w}_{f}t)\\ {my}^{\prime \prime }+C_{s}y^{\prime }+K_{s}y=-2m\Omega_{z}\times x^{\prime } \end{array}$$

where $w_{f}$ is the frequency of the drive signal, $F_{0}$ is the amplitude of the drive signal, the terms $C_{d}$, $C_{s}$ are the damping coefficients of the drive mode and the sense mode, the terms $K_{d}$, $K_{s}$ are the effective stiffness of the drive mode and the sense mode, $\Omega_{z}$ is the rotation rate along the gyroscope input axis.

The transfer function of the drive mode can be written as:

(10) H(s)=sms2+2ξdwds+wd2

where $\xi_{d}$ is damping ratio, and is defined as follows:

(11) $$\xi_{d}=\frac{C_{d}}{{2\mathrm{mw}}_{d}}$$

When the gyroscope is excited into the drive mode by the drive signal, the amplitude of the drive mode ($A_{d}$) is stabilized by an automatic gain control (AGC). The amplitude of the drive mode is illustrated in Equation (12):

(12) $$A_{d}=\frac{F_{0}}{K_{d}\sqrt{{\left({2\xi }_{d}\frac{1}{w_{d}}\right)}^{2}+{\left(\frac{1}{w_{f}}-\frac{w_{f}}{w_{d}{}^{2}}\right)}^{2}}}$$

The amplitude of the drive mode will be maximal when $w_{f}$ is equal to $w_{d}$. Thus, the amplitude of the drive mode with respect to the effective amplitude of the drive signal ($A_{0}$, drive amplitude) can be expressed as [21]:

(13) $$A_{d}=\frac{Q_{d}F_{0}}{{\mathrm{mw}}_{d}}=\frac{\sqrt{2}Q_{d}A_{0}}{{\mathrm{mw}}_{d}}$$

where Qd=12Cd is the quality factor of the drive mode.

The quality factor due to the air damping is proportional to the square root of the temperature and inversely proportional to the air pressure (P) in the gyroscope package according to the following equation [22]:

(14) $$Q_{d}=\frac{C\sqrt{k_{b}T}}{P}$$

where C is a constant associated with the gyroscope, $k_{b}$ is the Boltzmann constant, T is the operating temperature. As can be seen in Equations (13) and (14), the drive amplitude can be used to measure the operating pressure:

(15) $$P=\frac{C\sqrt{{2k}_{b}T}}{{\mathrm{mA}}_{d}w_{d}}A_{0}$$

The drive amplitude will change linearly with the operating pressure at a stable temperature because the drive-mode frequency is insensitive to the operating pressure. So, the drive amplitude can be used as a built-in barometer.

The linear dependence between the drive amplitude and the air pressure was demonstrated over a pressure range from 1 atmosphere to below 1 × 10^−4^ Pa. Table 2 lists the drive amplitudes at different pressures.

The pressure coefficient of the drive amplitude (PCA) was also obtained by fitting the data in Table 2. The coefficient is about 73.17 KPa/V.

The relationship between the drive amplitude and the gyroscope bias was measured using the same experiment setup. The air pressure in the vacuum chamber was pumped from 1 atmosphere to below 1 × 10^−4^ Pa, meanwhile the temperature was kept at the room temperature.

**Figure 5:**
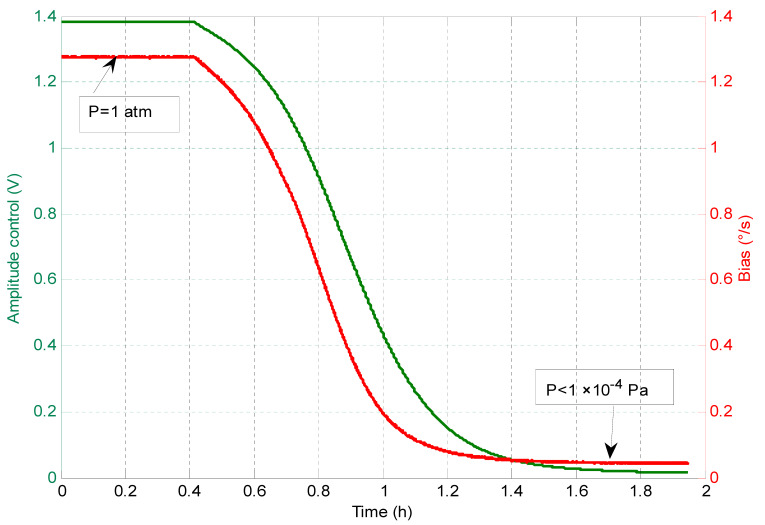
Measured bias drift vs. drive amplitude.

As shown in Figure 5, a positive bias-amplitude dependence was observed. A bias-amplitude dependence of 0.9 (°/s)/V was obtained by using the linear least squares fitting method again.

### 2.3. Bias Compensation Using Drive-Mode Parameters

**Figure 6:**
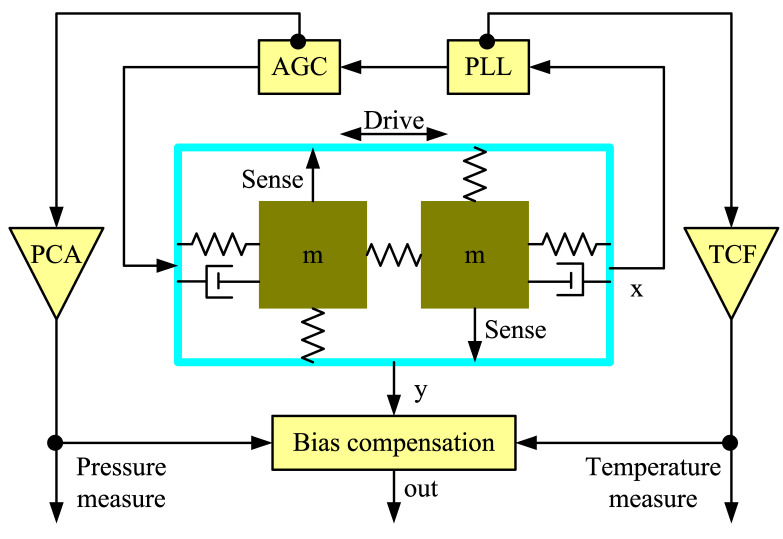
Signal processing for the bias compensation.

The drive-mode frequency is sensitive to the operating temperature. Moreover, the drive amplitude can be used as an embedded barometer. So, a novel control system using these drive-mode parameters for the MEMS gyroscope bias compensation is proposed. Figure 6 shows the signal processing for compensation of the bias drift.

The gyroscope drive-mode is controlled by a phase-locked loop (PLL) and an AGC loop. Similar control systems can be found in many other research studies [23,24,25]. The PLL is used to lock to the drive-mode frequency [26]. The instantaneous drive-mode frequency change is first correlated to the temperature using the measured TCF. Meanwhile, the change of the drive amplitude is correlated to the pressure using the measured PCA. Once the temperature and the pressure values are obtained, they are used to estimate and compensate the bias drift in real-time. Finally, the raw gyroscope output (y) is corrected by subtracting it with the sum of the estimated bias drifts. 

By monitoring the variations of the drive-mode parameters, the operating temperature and the air pressure in the gyroscope package can be known clearly, which provides a convenient means of obtaining the gyroscope health information.

## 3. Results and Discussion

### 3.1. Comprehensive Effect Verification

If the package is sufficiently hermetic, then the air pressure in the gyroscope package becomes proportional to the temperature [19]:

(16) $$P={\beta k}_{b}T$$

where β is the number density of molecules in the package. It means that there are dual effects on the gyroscope bias when the temperature changes.

**Figure 7:**
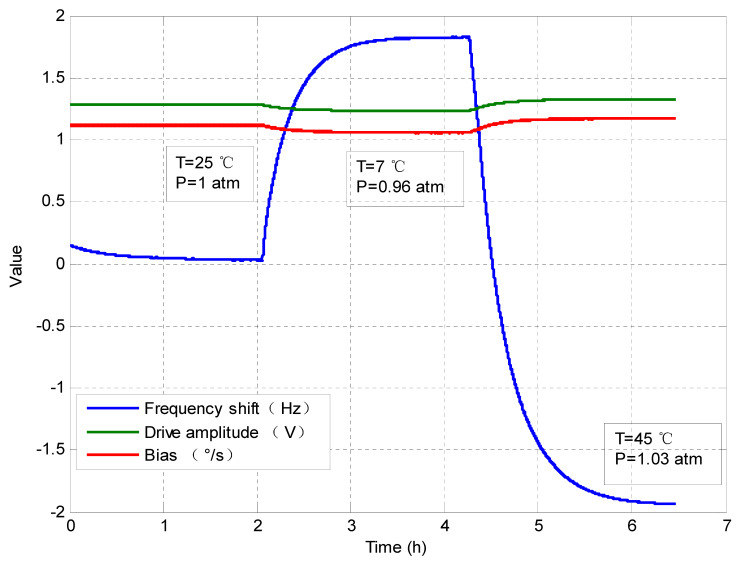
Dual effects on the gyro bias.

The dual effects of the temperature and the air pressure in the package on the gyroscope bias were tested, by subjecting an intact MEMS gyroscope to a temperature variation in the range from 7 °C to 45 °C. The drive-mode frequency shift, the drive amplitude, and the raw gyroscope output were shown in Figure 7. 

The air pressure in the gyroscope package marked in Figure 7 was obtained by using Equation (16) for the conversion. According to the dependences of the bias-frequency and the bias-amplitude, the change of the temperature and the air pressure should contribute to 0.037°/s and 0.121°/s of bias drift, respectively. In fact, the actual bias drift of the gyroscope was 0.148°/s. The error is about 0.01°/s, or 6.7%, due to the inevitable deviations of the model coefficients.

### 3.2. On-Orbit Experimental Demonstration

**Figure 8:**
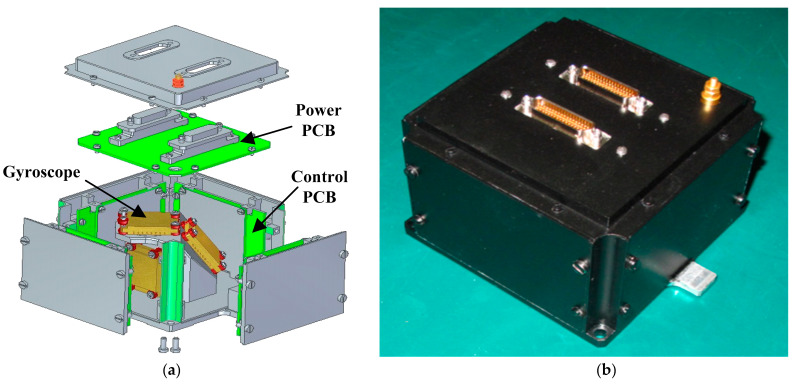
The MEMS rate sensor for space applications: (**a**) Mechanical architecture; (**b**) Photo of the MEMS sensor.

For the migration of the MEMS gyroscope technology to space, a MEMS rate sensor is developed and adopted for the on-orbit technology demonstration in the China’s new-generation communication satellite. The mechanical architecture is presented in Figure 8. The rate sensor is a cluster of four high accuracy MEMS-based gyros in a miniature package. In addition to the four MEMS-based gyros, the rate sensor is also comprised of the drive electronics, preamplifier circuitry for the sensor outputs, and digital conversion electronics.

**Figure 9:**
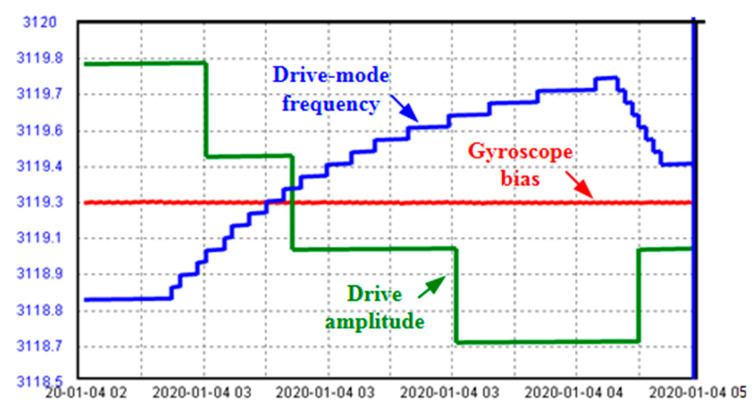
On-orbit data of the MEMS gyroscope.

The on-orbit data of one gyroscope, including the drive-mode frequency, the drive amplitude, and the gyroscope bias, as shown in Figure 9, are analyzed. The drive-mode frequency varies about 0.92 Hz in an hour, which is equivalent to a temperature variation of 9.2 °C using the TCF value for the conversion. Limited by the telemetry resources of the satellite, the resolution of the drive amplitude data is low, so the curve of the drive amplitude presents a step shape. As the drive-mode frequency increases, the drive amplitude decreases, which is in accordance with the laws of Equations (15) and (16). Although the temperature and the air pressure in the gyroscope package have changed, the gyroscope bias remains constant, revealing the robustness of the compensation approach.

## 4. Conclusions

In this paper, a MEMS gyroscope with atmosphere package was used to study the stability in the thermo-vacuum condition. The linear frequency–temperature dependence of −0.1 Hz/°C, as well as the linear amplitude–pressure dependence of 73.17 KPa/V, were obtained by experiment. A self-compensation of the gyroscope bias drift by utilizing its drive-mode parameters was proposed. Meanwhile, the air pressure and the temperature of the gyroscope were also measured. The meaningful on-orbit demonstration was conducted by a MEMS rate sensor using the same atmosphere-packaged MEMS gyroscopes. Although there was a deviation of about 6.7%, the demonstrated self-compensation method provides a path for atmosphere-packaged MEMS gyroscopes used for space applications with long-term bias stability.

Future works will focus on the analysis of other factors that influence the gyroscope bias and compensation algorithm optimization. We will also continue to monitor and analyze the data from the on-orbit MEMS rate sensor. When the technology demonstration is completed, the atmosphere-packaged MEMS gyroscopes can be equipped on many microsatellites.

## Figures and Tables

**Table 1:** Measured frequency–temperature dependence.

Temperature/°C	Drive-Mode Frequency/Hz
−20	3121.64
−5	3120.09
10	3118.58
35	3116.13
50	3114.61

**Table 2:** AGC drive amplitude vs. pressure.

Pressure/Pa	Drive Amplitude/V
101,000	1.38
80,000	1.11
10,000	0.15
2000	0.043
0.0001	0.015
